# Degrees of Testicular Atrophy Following Orchidopexy for Testicular Torsion

**DOI:** 10.7759/cureus.50543

**Published:** 2023-12-14

**Authors:** Nader Awad, Khalid Abdulaziz, Basma Malalla, Ali H Al Aradi, Ahmed A Al Rashed

**Affiliations:** 1 Urology, Salmaniya Medical Complex, Manama, BHR

**Keywords:** testicular atrophy, time to surgery, degree of torsion, testicular torsion, orchidopexy

## Abstract

Introduction

Testicular torsion is an urological emergency. It is a time-sensitive condition in which twisting of the spermatic cord and testicular blood supply occurs, causing acute onset severe scrotal pain. The incidence of testicular torsion is highest amongst prepubertal males; however, it can occur at any age. Every hour that passes from the onset of symptoms has been shown to decrease the salvageability rate of the torted testis. Another significant factor that impacts testicular salvage is the degree of torsion. Prompt surgical exploration of the scrotum and orchidopexy, if the testis is salvageable, is the mainstay of treatment. A major sequela following orchidopexy for torsion is the decrease in testicular volume. The aim of this study is to assess testicular volume loss post orchidopexy in patients who presented with testicular torsion, as well as to identify the significance of the degree of rotation and duration of torsion in post-fixation volume loss.

Methods

This is a retrospective study in which all patients who underwent scrotal exploration for a primary diagnosis of testicular torsion between June 1, 2016, to January 15, 2023, were reviewed. The information obtained included the patients’ demographics such as age, duration of symptoms, and laterality. Ultrasound images were reviewed for pre- and postoperative findings which included confirmation of testicular torsion as well as testicular volume measurements. Patients were excluded if they underwent an orchidectomy, had a diagnosis other than testicular torsion once scrotal exploration was done, or did not perform a follow-up scrotal ultrasound. Additionally, patients who underwent an orchidopexy for undescended testis earlier in life were also excluded. For statistical analysis purposes, degrees of testicular torsion and time to surgery were classified into mild, moderate, and severe.

Results

A total of 109 patient records were reviewed within the specific time frame. Of these, 47 patients were excluded as per the exclusion criteria mentioned previously, which gave us a sample size of 62 patients. Our findings showed that increasing severity of the degree of torsion as well as the time for surgery have statistically significant (p-value <0.05) effects on postoperative testicular volume loss. However, it was noted that time to surgery has a more pronounced effect on the mean volume loss than the degree of torsion. Moreover, the analysis also showed that, on average, with every additional hour from the onset of symptoms to surgery, the approximate volume loss is 0.15 ml. However, once time exceeds the 4.5-hour mark, the mean volume loss is 0.4 ml for each additional hour.

Conclusion

The current study indicates that earlier surgical intervention and correction of torsion are associated with enhanced preservation of postoperative testicular volume. Both the degree of torsion and time to surgery influence mean volume loss; however, time to surgery has a greater impact on the mean volume loss. These results highlight the importance of early diagnosis and intervention in cases of testicular torsion to minimize the risk of long-term testicular volume loss.

## Introduction

Acute scrotal pain is a relatively common emergency presentation, both in the primary care setting and in the emergency department, comprising approximately 0.5% of all emergency visits in the United States annually [1]. Testicular torsion is a true urological emergency in such cases of acute scrotal pain. Torsion is a time-sensitive condition in which twisting of the spermatic cord occurs and testicular blood supply compromise ensues, leading to acute onset severe scrotal pain [1]. Understanding the anatomy of the testicle is important in comprehending the pathophysiology of torsion. The tunica vaginalis is usually firmly attached to the posterolateral aspect of the testicle, and within it, the spermatic cord is not mobile. In cases where the attachment of the tunica vaginalis is high, the spermatic cord can twist more easily inside, leading to intravaginal torsion [1].

The incidence of testicular torsion is highest amongst prepubertal males; however, it can occur at any age [1]. Torsion typically presents with symptoms of acutely painful hemi-scrotum with a tender, elevated testis felt at a horizontal lie on clinical examination. As arterial blood supply is abruptly ceased, testicular detorsion is a race against time. Every hour that passes from the onset of symptoms has been shown to decrease the salvageability rate of the torted testis. Another significant factor that impacts testicular salvage is the degree of torsion. In most cases, 90-180 degrees of testicular rotation is capable of compromising testicular blood flow. Further degrees of torsion are rarer and significantly decrease the viability of the testes. The best salvage rates are seen within less than eight hours from the onset but become rare if more than 24 hours have elapsed [1,2].

A testicular ultrasound can be invaluable when available in a timely manner; however, it must not delay quick surgical intervention. It is considered the main adjunctive diagnostic modality beyond clinical examination. A color Doppler flow ultrasound for testicular torsion is approximately 93% sensitive and 100% specific, aiding in both diagnosis of testicular torsion as well as an assessment of testicular volume [3,4]. Once the diagnosis is made, the standard of care is immediate surgical intervention for testicular detorsion and bilateral orchidopexy if the testis is viable, or orchidectomy if necrosis has occurred [5].

The surgical management of testicular torsion depends on whether the testis is salvageable during surgical exploration. A black-colored testis is deemed necrotic, leading to orchidectomy, while a purple to whitish-pink-colored testis is considered viable, and bilateral orchidopexy is performed [5,6]. One major sequela following orchidopexy for torsion is the decrease in testicular volume. As testicular volumes decrease so does its ability for spermatogenesis and testosterone production. A poorly functioning testis can have long-term effects on patients in terms of fertility, as well as decreased libido, sexual dysfunction, and psychological impacts [6].

The aim of this study is to assess testicular volume loss post orchidopexy in patients who presented with testicular torsion as well as to identify the significance of the degree of rotation and duration of torsion in post-fixation volume loss.

## Materials and methods

All patients who underwent scrotal exploration for a primary diagnosis of testicular torsion between June 1, 2016, and January 15, 2023, were reviewed. All data were recorded from the hospital’s electronic database. Patients were excluded if they underwent an orchidectomy, had a diagnosis other than testicular torsion once scrotal exploration was done, or did not perform a follow-up scrotal ultrasound. Additionally, patients who were referred from other centers and had preoperative ultrasounds done outside our institute or who underwent an orchidopexy for undescended testis earlier in life were excluded.

The information obtained from the electronic files included the patients’ demographics such as age, duration of symptoms, and laterality. Images were reviewed for preoperative ultrasound findings, which included confirmation of testicular torsion as well as testicular volume measurements. Routine postoperative scrotal ultrasound is not done in our center unless patients have postoperative concerns that necessitate it. However, patients with at least six months of follow-up were contacted by phone and testicular volumes were measured by scrotal ultrasound. Testicular measurement was done using the formula of length (mm) × width (mm) × weight (mm) × 0.72. All scrotal ultrasounds were done using a GE LOGIQ E9 ultrasound machine (General Electric, Boston, Massachusetts, United States) using a linear 9 Hz transducer probe. All radiographic reporting was done by a senior radiology resident.

The local protocol in our center for a patient who presents with acute scrotal pain is simultaneous immediate shifting to ultrasound assessment and urological consultation. Once testicular torsion is suspected, patients are booked and shifted for surgery. The standard operative procedure practiced in our center is vertical scrotal incision starting on the affected side followed by delivery of the torted testis and assessment for viability and color followed by prompt detorsion while recording the degree of torsion. Once detorted, warm compressors are kept, and contralateral orchidopexy is performed. If the torted testis regains color and is visually viable, a dartos pouch is fashioned and 3-point fixation at 3, 6, and 9 o’clock is done using 3-0 Vicryl (Ethicon Inc., Raritan, New Jersey, United States). The dartos layer is also closed using 3-0 Vicryl, whereas skin closure is done using Rapide Vicryl. All surgeries were done under spinal anesthesia and performed by a senior urology resident.

For statistical analysis purposes, degrees of testicular torsion were classified into mild (90-180 degrees), moderate (180-360 degrees), and severe (>360 degrees). Furthermore, time for surgery was recorded in hours from the onset of symptoms until surgery start time and classified into mild (Less than four hours), moderate (four to six hours), and severe (more than six hours). A linear regression model was used to predict the relationship between testicular volume loss and the independent variables of degree of torsion and time to surgery. The equation used for the regression model was “Volume = β0 + β1 * Independent variable” in which β1 is the regression coefficient for the degree of torsion and β0 is the intercept. 

Additionally, given that time is an ordinal value, Spearman correlation coefficients were utilized to assess the relationship between the time of surgery and postoperative testicular volume loss. All statistical analysis was conducted using IBM SPSS Statistics for Windows, Version 29.0 (Released 2022; IBM Corp., Armonk, New York, United States), and 95% confidence intervals were calculated for the treatment’s success rates with p-values of < 0.05 considered statistically significant.

## Results

A total of 109 patient records were reviewed within the specific time frame. Forty-seven patients were excluded as per the exclusion criteria mentioned, which gave us a sample size of 62 patients. The patient and surgical parameters are given in Table 1.

**Table 1:** Patient and surgical parameters

	Mean + SD	Maximum Value	Minimum Value
Age (in years)	17.4 + 1.5 (32-14)	32	14
Duration (in hours)	5.8 + 1.2 (30-2)	30	2
Degree of Torsion	180 + 90 (90-540)	90	540

Our data showed that 29 (46.7%) patients presented with right-sided testicular torsion and 33 (53.3%) patients presented with left-sided testicular torsion. In terms of degrees of torsion, 19 patients (30.6%) had a mild degree whereas 28 (45.1%) patients and 15 (24.1%) patients had moderate and severe torsion respectively. The mean preoperative testicular volume on the unaffected side was 17.9 ml + 1.7 and the postoperative mean volume was 17.5 ml + 1.9. Comparatively, the mean preoperative volume of the affected testis was 18.5 ml + 2.1 whereas the mean postoperative volume was calculated for the different degrees of torsion and was as follows: mild 18.0 ml + 0.7, moderate 16.5 ml + 0.4, and severe 13.6 ml + 0.6.

In terms of time to surgery, 14 (22.5%) patients were considered within the mild group (< four hours), 31 (50%) patients and 17 (27.4%) patients were considered moderate (four to six hours), and severe (> six hours) respectively. The mean preoperative testicular volume in both the unaffected and affected side are identical to the previously mentioned volumes. However, the mean preoperative volume and the mean postoperative volume were calculated for the different times to surgery and were as follows: mild 17.8 ml + 0.5, moderate 16.2 ml + 0.3, and severe 12.9 ml + 0.8.

**Figure 1:**
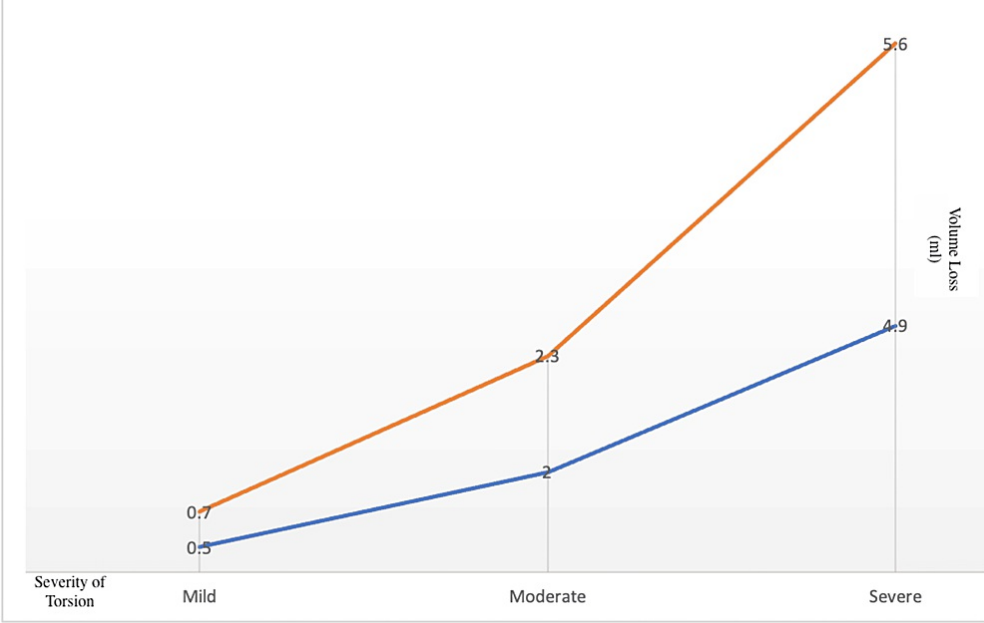
Mean postoperative volume loss in ml in affected testis with regard to severity of degree of torsion (blue curve) compared to that of time to surgery severity (orange curve)

Figure 1 illustrates how the mean testicular volume loss in ml increases as the severity of the degree of torsion and time to surgery increases. However, it can be noted that time to surgery (orange curve) has a more pronounced effect on the mean volume loss than the degree of torsion. 

Table 2 and Table 3 demonstrate the results of the linear regression models as they pertain to the severity of the degree of torsion and time to surgery with the postoperative testicular volume loss. Furthermore, the following results describe the relationship between the severity of the time to surgery and the postoperative volume loss in the affected testis as seen in Table 3. Increasing severity of the degree of torsion as well as the time for surgery have statistically significant (p-value <0.05) effects on postoperative testicular volume loss in ml.

**Table 2:** Severity of degree of torsion with postoperative testicular volume loss in affected testis

Degree of Torsion	Regression Coefficient	P-Value
Mild (90-180 degrees)	0.3	< 0.01
Moderate (180-360 degrees)	0.2	< 0.01
Severe (> 360 degrees)	0.1	< 0.05

**Table 3:** Severity of time to surgery with postoperative testicular volume loss in affected testis

Time to surgery	Regression Coefficient	P-Value
Mild (< 4 hours)	0.2	< 0.05
Moderate (4 -6 hours)	0.1	< 0.05
Severe (> 6hours)	0.1	< 0.01

Spearman correlation coefficients quantifying the relationship between postoperative testicular volume loss and severity of time to surgery were calculated and showed mild torsion: ρ = 0.65 (p < 0.05) moderate torsion: ρ = 0.52 (p < 0.05), severe torsion: ρ = 0.40 (p < 0.05). This is a positive correlation and signifies that as time to surgery increases, postoperative testicular volume loss tends to be higher. Moreover, the analysis showed that on average with every additional hour from the onset of symptoms to surgery, the approximate volume loss will be 0.15 ml; however, once time exceeds the 4.5-hour mark, the mean volume loss is 0.4 ml for every additional hour.

## Discussion

The management of testicular torsion is immediate surgical intervention with the aim of untwisting the spermatic cord and restoring blood supply to the affected testis as soon as possible [7]. Testicular salvageability is directly correlated to the time it takes to undergo surgical correction [8]. This has been best described in the systematic review done by Mellick et al., which concluded that if the surgical correction is conducted within less than six hours, the testis salvageability rate is around 97.2%, whereas this number decreases to 7.4% in patients who present after 48 hours [9]. Moreover, testicular spermatogenesis and hormonal production are proportionately affected by the total testicular volume; hence, testicular volume following repair is an important factor for determining postoperative testis function. Mellick et al. also noted a permanent effect on testicular spermatogenesis and endocrine function, which occurs once the period of torsion exceeds eight hours [9]. These findings align with what we observed in our study, which showed that the longer the time increases, the higher the volume loss. However, when comparing our findings to the systematic review, we notice that significant postoperative volume loss can occur in patients who underwent orchidopexy as early as four hours after the onset of symptoms.

Furthermore, we demonstrated that time is clearly the more important determinant of postoperative testicular volume loss, as seen in Figure 1. However, the effect of the degree of torsion cannot be understated. This is clear in the comparative graph in Figure 1, which demonstrates the different mean volume losses between the different severity grades of time and degree of torsion. Additionally, our regression model values show a significant statistical correlation between higher degrees of testicular torsion and increased postoperative volume loss. Howe et al., in their study conducted in 2017, also looked into the degree of twisting and its clinical significance on testicular torsion outcomes [10]. They concluded that when the spermatic cord undergoes more than 360 degrees of twisting, there is up to a 25% chance of orchiectomy. However, in the current study, we were more concerned with the salvageable testicular volume, and to add to their findings, we conclude that a severe degree of torsion (360 degrees and more) was statistically (p-value <0.05) associated with the highest amount of volume loss where we saw a mean volume loss of around 4.9 ml.

Additionally, in a study conducted in 2016 by Dias Filho et al., they reviewed the spermatic cord rotation effect on the outcomes of intravaginal testicular torsion [11], and demonstrated similar findings to the current study. They concluded that presentation delay is the major factor in determining surgical outcomes. However, the degree of spermatic cord rotation exerts a multiplicative effect on time to surgery and increases the chances of orchiectomy. Concurrently, they also found that both presentation delay, as well as degree of torsion, were inversely proportional to chances of orchidopexy [11]. However, they did not study the exact effect that these variables have on post-orchidopexy volumes as was done in the present study. 

When looking at the time to surgery as an independent factor for testicular volume loss, it can be seen from Figure 1 that there appears to be a directly proportionate relationship in which more time leads to higher volume loss. Although the relationship is directly proportionate [12], it is not particularly linear. This was noted when our statistical analysis showed that the mean testicular volume loss per hour appears to be significantly higher once the time to surgery exceeds 4.5 hours. Comparatively, the total time average volume loss from the onset of symptoms to surgical correction is only 0.15 ml. This signifies within four to five hours from the onset of symptoms, significant volume loss should be expected even if orchidopexy is done, and the patient should be counseled accordingly to manage the post-operative expectations.

Furthermore, another study published in 2015, which investigated the factors influencing testicular atrophy following torsion, showed that if the time to surgery exceeds 24 hours, then 91% of patients are expected to develop significant testicular atrophy postoperatively [13]. Our findings are consistent with this; however, we observed that the onset of significant atrophy actually occurs around four hours from the onset of symptoms. This underlines the importance of immediate diagnosis to prevent long-term permanent damage.

The psychological and clinical impacts of post-orchidopexy testicular volume loss cannot be understated. In addition to concerns that arise regarding future fertility, the masculine perception of the individual can be affected by the physical size of the testis, leading to feelings of inadequacy and even low self-esteem [14,15]. It is well established that libido is considerably affected by stress and can lead to performance anxiety [15]. Patients who suffer from abnormally small testicular sizes or particularly from an uneven-appearing scrotum might experience an added psychological effect leading to decreased ability to perform sexually and even a tendency to avoid sexual intercourse due to fear of being stigmatized [16].

Limitations of our study include the fact that ultrasound imaging was not performed by the same radiologist, which can lead to differences in calculating testicular volumes. This was difficult to address given that the presentation of torsion is an acute emergency and imaging needs to be done immediately by the on-call senior radiologist without the possibility of delay. Furthermore, the postoperative testicular volume was measured at approximately six-month intervals due to resource and schedule limitations. Further imaging at longer intervals, such as one and two years postoperatively, can help further assess the effects torsion has on testicular volume.

## Conclusions

Our study indicates that earlier surgical intervention and correction of torsion are associated with enhanced preservation of postoperative testicular volume. Both the degree of torsion and time to surgery influence mean volume loss; however, time to surgery shows a greater effect on mean volume loss. These results highlight the importance of early diagnosis and intervention in cases of testicular torsion to minimize the risk of long-term testicular volume loss.
